# Prognostic Value of the Lung Immune Prognostic Index (LIPI) in Patients with Renal Cell Carcinoma: A Retrospective Cohort Study

**DOI:** 10.3390/jcm15031188

**Published:** 2026-02-03

**Authors:** Beyza Ünlü, Hacer Demir, Sena Ece Davarcı, Yaşar Culha, Meltem Baykara

**Affiliations:** 1 Medical Oncology Department, Afyonkarahisar Health Science University, 03030 Afyonkarahisar, Turkey; drhacerdemir@gmail.com (H.D.); beyaaz3@hotmail.com (S.E.D.); baykara.meltem@gmail.com (M.B.); 2 Medical Oncology Department, Sivas Numune Hospital, 58060 Sivas, Turkey; drjasar@hotmail.com

**Keywords:** LIPI, renal cell carcinoma, prognosis, IMDC, MSKCC, survival, retrospective

## Abstract

**Background:** The Lung Immune Prognostic Index (LIPI) has recently emerged as a novel prognostic biomarker in several malignancies, particularly in patients receiving immunotherapy. However, its role in renal cell carcinoma (RCC), especially in non-metastatic and tyrosine kinase inhibitor (TKI)-treated patients, remains unclear. **Methods:** In this retrospective cohort study, 153 patients diagnosed with RCC between 2012 and 2024 were analyzed. Prognostic scores including LIPI, International Metastatic RCC Database Consortium (IMDC), and Memorial Sloan Kettering Cancer Center (MSKCC) scores were calculated. The patients were stratified into risk groups (good, intermediate, and poor) based on these scores. Survival analyses were performed using Kaplan–Meier and Cox regression methods. Correlations between scoring systems were assessed using Pearson’s correlation. **Results:** The median follow-up was 29.1 months. A total of 55 (35.9%) patients had metastases at diagnosis. LIPI was significantly associated with overall survival (OS), progression-free survival (PFS), and disease-free survival (DFS) (*p* < 0.05). In the multivariate Cox analysis, LIPI remained an independent prognostic factor for OS and PFS. Strong positive correlations were found between LIPI and both IMDC and MSKCC scores (r > 0.6, *p* < 0.001). Notably, LIPI demonstrated prognostic relevance even in patients treated with TKIs. **Conclusions:** LIPI is a simple and accessible prognostic tool that provides significant survival stratification in RCC patients. Its predictive utility extends beyond immunotherapy cohorts, indicating potential value in broader RCC management. Integration of LIPI into current prognostic models may improve individualized treatment approaches.

## 1. Introduction

Renal cell carcinoma (RCC) accounts for approximately 2% of all cancers and cancer-related deaths worldwide [1]. It constitutes more than 90% of all kidney tumors [2]. While localized RCC can be treated with surgery alone, systemic therapy is required for metastatic RCC. However, RCC is resistant to standard chemotherapy. Over the past decade, anti-angiogenic agents targeting vascular endothelial growth factor (VEGF) and its receptors, mTOR inhibitors, and immune checkpoint inhibitors have improved clinical outcomes in RCC and have been incorporated into treatment algorithms [3].

Carcinogenesis arises from an imbalance between cell proliferation and apoptosis. Various chronic inflammatory conditions can render susceptible cells more prone to neoplastic transformation. Reactive nitrogen and oxygen species may directly oxidize DNA or impair DNA repair mechanisms. As a result, the longer chronic inflammation persists, the greater the risk of carcinogenesis is considered to be [4,5].

There are studies indicating that inflammatory indices such as albumin, C-reactive protein (CRP), and the derived neutrophil-to-lymphocyte ratio (dNLR) are associated with prognosis in malignancies, and that elevated LDH levels are related to tumor metastasis and proliferation in malignancies [6,7,8,9]. Therefore, combining these indices may provide more accurate prognostic information and thus the Lung Immune Prognostic Index (LIPI) was developed. It was initially found to be associated with immunotherapy response in patients with non-small cell lung cancer [10,11,12]. Subsequently, it was evaluated in resected esophageal squamous cell carcinoma and found to be associated with prognosis [13]. However, the literature on the prognostic role of LIPI in renal cell carcinoma remains limited.

Although LIPI is a novel prognostic indicator based on systemic inflammatory markers, the available data on its prognostic power in RCC is limited, and most studies have been conducted in metastatic populations. It therefore remains unclear whether LIPI retains prognostic relevance in real-world RCC cohorts that include patients treated predominantly with tyrosine kinase inhibitors and those with non-metastatic disease.

Moreover, the biological relevance of systemic inflammation-based scores such as LIPI in surgically treated, early-stage disease is uncertain as tumor burden and systemic inflammatory response are expected to be lower in this setting. In this study, we aimed to evaluate the correlation between the LIPI score and overall survival in both metastatic and non-metastatic RCC patients, and to analyze the correlation between LIPI, MSKCC, and IMDC scores. Secondary objectives included exploratory analyses of its association with progression-free survival and disease-free survival, acknowledging the limited evidence supporting these endpoints.

## 2. Materials and Methods

This single-center cohort study was conducted retrospectively using data from patients diagnosed with renal cell carcinoma (RCC) and treated/followed up between 2012 and 2024 at the Department of Medical Oncology, Afyonkarahisar Health Sciences University.

Patient characteristics including age, sex, histological subtype, tumor grade, metastatic status at diagnosis, surgical approach, systemic treatment lines, and follow-up durations were analyzed. Patients with missing data, inaccessible records, or multiple primary malignancies were excluded, resulting in a total of 153 patients included in the study. For patients who were non-metastatic at diagnosis and underwent surgery, disease-free survival (DFS) was also evaluated.

Overall survival (OS) was defined as the time from diagnosis to death or last follow-up. Progression-free survival (PFS) was defined as the time from the initiation of systemic therapy to disease progression or death. DFS was defined as the time from surgery to recurrence or death.

The LIPI score was calculated using serum lactate dehydrogenase (LDH) levels and derived neutrophil-to-lymphocyte ratio (dNLR), which was calculated as neutrophil/(leukocyte − neutrophil). One point was assigned for each of the following: LDH above the upper limit of normal and dNLR > 3. Based on the total score, patients were classified into three LIPI risk groups: 0 (good), 1 (intermediate), and 2 (poor). MSKCC and IMDC scores were also calculated according to standard criteria.

All survival analyses were performed using the Kaplan–Meier method, and differences between groups were assessed using the log-rank test. Variables found to be significant in the univariate analysis were included in the multivariate Cox regression model. Since the IMDC, MSKCC, and LIPI scores contain overlapping prognostic parameters, they were analyzed in separate models to avoid multicollinearity. Each model additionally included sex, presence of metastasis at diagnosis, and Karnofsky performance status.

Pearson correlation analysis was used to assess relationships between the scoring systems, and Fisher’s exact test was used to evaluate associations between categorical variables. A *p*-value of <0.05 was considered statistically significant. All statistical analyses were performed using IBM SPSS Statistics version 20 (IBM Corp., Armonk, NY, USA).

A post hoc power analysis was conducted to evaluate the adequacy of the sample size. Assuming a hazard ratio of 3.0 between LIPI risk groups, with α = 0.05 and an event rate of 50%, the statistical power was calculated to be 91.2%, indicating sufficient power for survival analysis.

## 3. Results

A total of 153 patients were included in the study. The median age was 63 years (range: 34–85), and 97 patients (63.4%) were male. A performance score above 80 was observed in 92.8% of the patients. Comorbidities were present in 54.9% of the patients. At the time of diagnosis, 110 patients (71.9%) had undergone radical nephrectomy. The most common histological subtype was clear cell carcinoma (72.5%), followed by papillary (11.1%) and chromophobe (5.9%) subtypes. Grade 2 and 3 tumors were the most frequent. At diagnosis, 35.9% of the patients had metastatic disease, with the lung being the most common site of metastasis (26.8%).

First-line metastatic treatment was administered to 78 patients (51.0%). The most frequently used agents were pazopanib (50.0%), sunitinib (32.1%), and cabozantinib (14.1%). Disease progression occurred in 68.3% of these patients. Second-line therapy was given to 33 patients (21.6%), which included nivolumab (63.6%), everolimus (27.3%), and axitinib (6.1%). Ten patients did not receive second-line treatment due to poor performance status, refusal of treatment, or loss to follow-up. Progression occurred in 45.4% of the patients receiving second-line therapy. Third-line treatment was administered to 13 patients (8.5%) (Table 1).

Although 153 patients were included, LIPI scores were only available for 143 patients due to missing baseline laboratory data in 10 individuals. Among the patients, 21.7% were in the good risk group (score 0), 60.8% were in the intermediate group (score 1), and 17.5% were in the poor risk group (score 2). Due to missing data, MSKCC and IMDC scores could only be calculated for 63 patients each (Table 1).

The median overall survival (OS) in the entire cohort was 70.2 ± 10.8 months, while it was 33.7 ± 8.0 months in metastatic patients. Grouping metastatic patients by sex, metastatic status at diagnosis, Karnofsky performance status, MSKCC, IMDC, and LIPI scores found statistically significant differences in OS between groups. No significant association was found between OS and comorbidities, tumor histology, tumor grade, or type of first-line therapy (*p* = 0.656, 0.420, and 0.105, respectively). The median OS was 33.2 months with pazopanib, 51.7 months with sunitinib, and 23.4 months with cabozantinib, but no statistically significant difference in OS was observed between treatment groups (*p* = 0.960) (Table 2).

Because IMDC, MSKCC, and LIPI scores share similar prognostic parameters, each was analyzed in separate models to avoid multicollinearity. Multivariate Cox regression analyses were conducted in three separate models, each including either IMDC, MSKCC, or LIPI scores, along with sex, presence of metastasis at diagnosis, and Karnofsky performance status. Good risk groups were taken as reference. In all three models, poor-risk groups according to the LIPI, MSKCC, and IMDC scores were associated with significantly worse overall survival. Patients with high LIPI scores had significantly increased risk of death compared to those with low scores (HR: 0.150, 95% CI: 0.056–0.397; *p* < 0.001). The intermediate-risk group was also statistically significant (HR: 0.411; *p* = 0.007). Presence of metastasis at diagnosis and Karnofsky performance status were independent predictors of mortality in the LIPI and IMDC models. Sex was not found to be an independent prognostic factor in any of the models. LIPI score significantly reduced survival in both intermediate and poor-risk groups, suggesting it is a strong prognostic factor (Table 3).

Among the 85 metastatic patients, disease progression after first-line treatment occurred in 43 patients (50.6%). The median progression-free survival (PFS) was 10.8 ± 2.9 months (95% CI: 5.0–16.7). No significant associations were found between PFS and sex, comorbidities, tumor grade, MSKCC score, or LIPI score (*p* values: 0.424, 0.321, 0.457, 0.540, and 0.776, respectively). However, tumor histology, metastatic status at diagnosis, and IMDC score significantly affected PFS in the univariate analysis. The significant factors identified in the univariate analysis did not retain significance in the multivariate analysis. No significant difference in PFS was observed between LIPI and the other scoring systems (Table 4).

Of the 43 patients who progressed, 33 received second-line therapy. Ten patients did not receive treatment due to poor general condition or refusal. The median PFS after second-line therapy was 11.6 ± 4.1 months (95% CI: 3.4–19.7). Among the groups based on LIPI scores, PFS was 36.0 months in the good group, 7.5 months in the intermediate group, and 8.1 months in the poor group, but these numerical differences did not reach statistical significance (*p* = 0.154). Although treatment response and disease control rates numerically improved in the low LIPI score group, they were not statistically significant. The most common second-line therapies were nivolumab (63.6%), everolimus (27.3%), and axitinib (6.1%). No significant difference in PFS was observed between different treatment regimens (*p* = 0.189). Similarly, no significant relationship was found between LIPI score and objective response rate (ORR) or disease control rate (DCR) in both first- and second-line treatments (Table 5).

In the patients without metastasis at diagnosis, the median disease-free survival (DFS) was 129 ± 24.8 months (95% CI: 81.0–178.5). Low tumor grade and increasing IMDC score were significantly associated with DFS (*p* = 0.033 and 0.010, respectively). No patients were classified as poor risk based on IMDC scoring in this subgroup. The median DFS was 36.8 months in the IMDC good risk group and 78.4 months in the intermediate risk group. Sex, comorbidities, histologic type, LIPI and MSKCC scores were not associated with DFS (*p* values: 0.661, 0.347, 0.058, 0.743, and 0.376, respectively).

To assess the relationships among the three prognostic scores used in patients with RCC (LIPI, MSKCC, and IMDC), Pearson correlation analysis was performed. A positive correlation was found between LIPI and MSKCC (r = 0.420), and LIPI and IMDC (r = 0.361). A strong positive correlation was also observed between MSKCC and IMDC (r = 0.723).

## 4. Discussion

Renal cell carcinoma (RCC) is the most common form of kidney tumors and accounts for approximately 2–3% of all cancers. It is more common in males, and the average age at diagnosis tends to be older [1]. Our study also reflected a male predominance. Histopathologically, clear cell carcinoma is the most common subtype, followed by papillary and chromophobe subtypes, which is consistent with our findings.

Approximately 30% of RCCs are metastatic at diagnosis, and among patients with localized or locally advanced disease, up to 30% may develop metastasis later. The lungs are the most common site of metastasis [14]. In our study, the metastatic rate at diagnosis was 35.9%, with the lungs being the most frequent metastatic site, in line with existing literature.

The RCC patient population in our study was generally similar to previously reported groups in terms of clinical characteristics and histological subtype distribution. Based on this, the prognostic impact of the LIPI, MSKCC, and IMDC scoring systems on survival and treatment response was analyzed. MSKCC and IMDC scores are widely used for prognostic stratification in metastatic RCC, but they rely on different parameters compared to LIPI. Since LIPI only includes two biochemical and hematological markers, it offers a fast and cost-effective tool in clinical practice. In our study, the LIPI score emerged as a prognostic indicator, particularly for overall survival (OS). However, it did not show a significant association with progression-free survival (PFS), similar to MSKCC and IMDC scores in this cohort. This may suggest that LIPI reflects overall systemic vulnerability rather than tumor-specific progression.

A unique feature of our study is that it included non-metastatic RCC patients and evaluated the effects on disease-free survival (DFS). There are very few studies investigating the association between LIPI and DFS. In our study, no significant relationship between LIPI and DFS was observed, possibly due to the smaller role of systemic inflammation markers in non-metastatic settings. This finding suggests that inflammation-based scores such as LIPI may have limited biological relevance in surgically treated early-stage disease.

LIPI was first evaluated in metastatic non-small cell lung cancer (NSCLC) patients treated with immune checkpoint inhibitors [15]. Several studies have reported its prognostic value in NSCLC and small cell lung cancer [10]. Recent evidence suggests that the prognostic value of the Lung Immune Prognostic Index (LIPI) is not limited to the metastatic setting. In a study by Hoshina et al. that included patients with pathological T3 renal cell carcinoma, a higher preoperative LIPI score was significantly associated with worse overall survival, suggesting that LIPI may also have prognostic relevance in non-metastatic disease [16]. Outside of the lung, Feng et al. demonstrated that LIPI is an independent prognostic index for cancer-specific survival in resected esophageal carcinoma [13]. A recent study published in the European Journal of Cancer validated LIPI as an independent prognostic factor for OS and PFS in metastatic RCC, even after adjusting for IMDC scores [17]. Unlike that study, our research included both metastatic and non-metastatic RCC patients, allowing us to explore the prognostic value of LIPI for DFS—an aspect rarely addressed in the literature. Although no significant association was found, this exploratory analysis provides preliminary data for future studies. Additionally, we performed a comprehensive evaluation of all three scoring systems (LIPI, MSKCC, and IMDC), analyzing their correlations within the same patient cohort.

As LIPI is based on inflammation, previous studies have explored its association with response to immunotherapy. Poor LIPI scores have been linked to lower immunotherapy response rates [18]. In our cohort, the number of immunotherapy-treated patients was limited, and no statistically significant predictive association could be demonstrated. However, the observation that LIPI retained prognostic value for overall survival even in predominantly TKI-treated patients may warrant further investigation.

We observed positive correlations among LIPI, IMDC, and MSKCC scores, suggesting that these scoring systems often identify similar risk groups. However, this analysis was limited by the high proportion of missing IMDC and MSKCC data, and therefore should be interpreted cautiously.

In conclusion, although our study has limitations, such as its retrospective design, single-center setting, heterogeneous population, and missing prognostic data, it provides real-world evidence regarding the prognostic role of LIPI in RCC. These findings should be considered hypothesis-generating and require validation in larger, prospective, and more homogeneous cohorts.

## 5. Conclusions

In this retrospective study, the LIPI score was shown to be an independent prognostic marker for overall survival in renal cell carcinoma (RCC), even among patients primarily treated with tyrosine kinase inhibitors. While not significantly associated with progression-free or disease-free survival, LIPI’s link to early mortality and systemic inflammation highlights its clinical relevance. Positive correlations between LIPI, MSKCC, and IMDC scores support their complementary prognostic roles. Despite its limitations, this study is among the first to assess LIPI’s impact on disease-free survival alongside other scoring systems, suggesting its utility as a simple, accessible prognostic tool in RCC.

## Figures and Tables

**Table 1:** Clinical characteristics of patients.

Variable	n (%)
Median age (range) in years	63 (34–85)
Gender (No. (%))	
Female	56 (36.6%)
Male	97 (63.4%)
Karnofsky performance status (No. (%))	
>80	142 (92.8%)
<80	11 (7.2%)
Comorbidity (No. (%))	
Yes	84 (54.9%)
No	69 (45.1%)
Surgery (No. (%))	
Radical nephrectomy	110 (71.9%)
Nephron-sparing surgery	16 (10.5%)
No	27 (17.7%)
Histologic type (No. (%))	
Clear cell carcinoma	111 (72.5%)
Papillary carcinoma	17 (11.1%)
Chromophobe cell carcinoma	9 (5.9%)
Other	16 (10.5%)
Tumor grade (No. (%))	
1	11 (7.2%)
2	44 (28.8%)
3	37 (24.2%)
4	17 (11.1%)
Unknown	44 (28.8%)
Metastasis at diagnosis (No. (%))	
Yes	55 (35.9%)
No	98 (64.1%)
Metastasis location (No. (%))	
Lung	41(26.8%)
Non-regional lymph node	37 (24.2%)
Bone	22 (14.3%)
Surrenal	16 (10.5%)
Liver	13 (8.5%)
Brain	3 (2.0%)
Metastatic treatment (No. (%))	
First line	78 (51.0%)
Second line	33 (21.6%)
Third line	13 (8.5%)
Metastatic First Line Treatment (No. (%))	
Pazopanib	39 (50.0%)
Sunitinib	25 (32.1%)
Cabozantinib	11 (14.1%)
Sorafenib	1 (1.3%)
Ipilimumab–Nivolumab	1 (1.3%)
Nivolumab	1 (1.3%)
Metastatic Second Line Treatment (No. (%))	
Nivolumab	21 (13.7%)
Everolimus	9 (5.9%)
Axitinib	2 (1.3%)
Sunitinib	1 (0.7%)
Metastatic Third Line Treatment (No. (%))	
Axitinib	8 (61.5%)
Cabozantinib	3 (23.1%)
Pazopanib	1 (7.7%)
Everolimus	1 (7.7%)
LIPI Score (No. (%))	
Favorable	31 (20.3%)
Intermediate	87 (56.9%)
Poor	25 (16.3%)
Unknown	10 (6.5)
MSKCC Score (No. (%))	
Favorable	9 (5.8%)
Intermediate	28 (18.2%)
Poor	26 (16.9%)
Unknown	91 (59.1%)
IMDC Score (No. (%))	
Favorable	12 (7.8%)
Intermediate	18 (11.7%)
Poor	33 (21.4%)
Unknown	91 (59.1%)

**Table 2:** Univariate overall survival analyses in metastatic patients.

Variable	Median Overall Survival (m)	Log-Rank X^2^	*p*-Value
Gender		5.678	0.017
Female	55.63 ± 16.194 [23.8–87.3] (95% CI)		
Male	26.57 ± 6.3 [14.0–39.0] (95% CI)		
Comorbidity		0.199	0.656
Yes	36.7 ± 11.1 [14.8–58.5] (95% CI)		
No	33.2 ± 15.2 [3.3–63.0] (95% CI)		
Histologic Type		3.899	0.42
Clear cell carcinoma	33.733 ± 10.9 [12.3–55.1] (95% CI)		
Papillary carcinoma	33.2 ± 17.5 [0.0–67.2] (95% CI)		
Chromophobe cell carcinoma	230.933		
Other	11.467 ± 2.7 [5.9–16.9] (95% CI)		
Tumor Grade			0.105
1	9.833 ± [9.2–19.8] (95% CI)		
2	70.4 ± 56.543 [0.0–181.25] (95% CI)		
3	52.0 ± 9.9 [32.5–71.4] (95% CI)		
4	49.533 ± 36.7 [0.0–121.4] (95% CI)		
Metastasis at Diagnosis		30.372	<0.001
Yes	15.067 ± 3.6 [7.8–22.2] (95% CI)		
No	79.4 ± 20.1 [39.9–118.8] (95% CI)		
Karnofsky Performance Status		25.034	<0.001
<80	2.833 ± 0.5 [1.7–3.9] (95% CI)		
>80	41.9 ± 9.0 [24.0–59.7] (95% CI)		
Metastatic First-Line Treatment		0.299	0.960
Sunitinib	51.7 ± 17.9 [16.6–86.8] (95% CI)		
Pazopanib	33.2 ± 7.2 [199.0–47.3] (95% CI)		
Cabozantinib	23.4 ± 7.6 [8.3–38.4] (95% CI)		
Other	-		
LIPI Score		19.271	<0.001
Favorable	175.233		
Intermediate	33.733 ± 14.1 [5.9–61.4] (95% CI)		
Poor	8.233 ± 1.6 [4.9–11.5] (95% CI)		
MSKCC Score		29.306	<0.001
Favorable	79.4 ± 24.7 [30.9–127.8] (95% CI)		
Intermediate	47.633 ± 13.6 [20.8–74.4] (95% CI)		
Poor	8.6 ± 3.0 [2.6–14.5] (95% CI)		
IMDC Score		22.535	<0.001
Favorable	55.633 ± 6.6 [42.5–68.7] (95% CI)		
Intermediate	52.0 ± 15.4 [21.6–82.3] (95% CI)		
Poor	7.6 ± 2.2 [3.2–11.9] (95% CI)		

**Table 3:** Multivariate Cox regression analysis of overall survival in metastatic patients was performed based on three separate models constructed using IMDC, MSKCC, and LIPI scores.

Variable	Model	HR (Exp(B))	95% CI (Lower–Upper)	*p*-Value
Gender	IMDC-based model	1.025	-	0.951
	MSKCC-based model	1.343		0.476
	LIPI-based model	1.121		0.730
Metastasis at diagnosis	IMDC-based model	2.841	1.103–7.315	0.030
	MSKCC-based model	2.499	0.916–6.818	0.074
	LIPI-based model	4.528	2.229–9.197	<0.001
Karnofsky performance status	IMDC-based model	0.279	0.121–0.645	0.003
	MSKCC-based model	0.599	0.252–1.423	0.246
	LIPI-based model	0.387	0.181–0.828	0.014
IMDC (intermediate)	IMDC-based model	0.627	0.211–1.862	0.400
IMDC (poor)	IMDC-based model	0.388	0.181–0.832	0.015
MSKCC (intermediate)	MSKCC-based model	0.297	0.080–1.105	0.070
MSKCC (poor)	MSKCC-based model	0.345	0.169–0.704	0.003
LIPI (intermediate)	LIPI-based model	0.150	0.056–0.397	<0.001
LIPI (poor)	LIPI-based model	0.411	0.214–0.787	0.007

**Table 4:** Kaplan–Meier and Cox regression analysis results of variables associated with PFS at the subgroup level.

Variable	mPFS (m)	95% CI (Lower–Upper)	*p* (Kaplan–Meier)	HR (Cox)	*p* Value (Cox)
Histologic Type			0.022		
Clear cell carcinoma	11.2	2.1–20.3		ref	
Papillary carcinoma	7.5	3.8–11.2		0.359	0.348
Cromophobe cell	4.0	–		0.561	0.649
Other	19.5	0–51.0		8.087	0.201
Metastasis at Diagnosis			0.009		
No	19.5	0–44.3		ref	
Yes	9.5	4.6–14.4		0.395	0.178
IMDC Score			0.048		
Favorable	59.6	0–123.4		ref	
Intermediate	11.2	2.3–20.2		0.646	0.626
Poor	9.1	4.8–13.4		0.776	0.582
LIPI Score			0.776		
Favorable	6.6	0–13.6		-	-
Intermediate	11.2	3.3–17.7		-	-
Poor	17.5	3.1–31.9		-	-
MSKCC Score			0.54		
Favorable	16.8	0–37.6		-	-
Intermediate	11.2	0–23.9		-	-
Poor	9.5	4.9–14.0		-	-

**Table 5:** ORR and DCR score in 1st- and 2nd-line therapy between groups based on LIPI.

	Good LIPI	Intermediate LIPI	Poor LIPI	*p*-Value (Fisher’s Exact Test)
ORR-1 (CR + PR)	50.0%	44.7%	33.3%	0.415
DCR-1 (CR + PR + SD)	78.6%	68.1%	53.3%	1.000
ORR-2 (CR + PR)	54.5%	50.0%	44.4%	1.000
DCR-2 (CR + PR + SD)	90.9%	75.0%	50.0%	0.243

## Data Availability

The datasets used and/or analyzed during the current study are available from the corresponding author upon reasonable request.
